# MoLEP—Co-creating a *Mycobacterium leprae* transmission interruption program for the Morogoro region, Tanzania

**DOI:** 10.1371/journal.pntd.0013507

**Published:** 2025-09-19

**Authors:** Tanja Barth-Jaeggi, Guy de Coulon, Issa Garimo, John Msaki, Liberate Mleoh, Riziki Kisonga, Sunil Modali, Shaishav Panchal, Shigela Marco Njebele, Shamez Sunderji, Kihulya Mageda, Peter Steinmann

**Affiliations:** 1 Swiss Tropical and Public Health Institute, Allschwil, Switzerland; 2 University of Basel, Basel, Switzerland; 3 Novartis Kenya Ltd., Nairobi, Kenya; 4 German Leprosy and Tuberculosis Relief Association Tanzania, Dar es Salaam, Tanzania; 5 National Tuberculosis and Leprosy Programme, Dodoma, Tanzania; 6 Novartis Pharmaceuticals, London, United Kingdom; 7 Novartis Healthcare Pvt. Ltd., Hyderabad, India; 8 President’s Office – Regional Administration and Local Government, Dodoma, Tanzania; London School of Hygiene and Tropical Medicine, UNITED KINGDOM OF GREAT BRITAIN AND NORTHERN IRELAND

## Abstract

The global goal for leprosy elimination is the interruption of *Mycobacterium leprae* transmission, resulting in zero new leprosy patients. In alignment with this objective, Tanzania’s updated national leprosy strategy emphasizes early detection, systematic contact tracing, post-exposure prophylaxis (PEP) with single-dose rifampicin (SDR), and integration of services into primary health care. However, the feasibility of accelerating *M. leprae* transmission interruption in high-burden areas with this strategy has yet to be demonstrated. In 2024, the Morogoro Leprosy Elimination Program (MoLEP) was launched in the country’s most endemic region. Developed through a collaborative process, MoLEP aligns global practices with local conditions and needs. Key interventions include training frontline health workers and expanding contact tracing with SDR-PEP. Furthermore, targeted activities will respond to high transmission areas and the detection of child cases, which serve as indicators of recent transmission. Strengthening the drug supply chain is also a priority. MoLEP will generate critical data on the feasibility and effectiveness of these interventions in accelerating progress toward elimination. The implementation is guided by a comprehensive monitoring and evaluation framework, alongside clearly defined governance structures, to facilitate evidence-informed decision-making. Findings are expected to guide strategic scaling and replication in other high-burden regions across Tanzania and beyond.

## Background

Post-exposure prophylaxis (PEP) with single-dose rifampicin (SDR) reduces the risk of leprosy among contacts. A large study in Bangladesh (COLEP) showed 57% reduction in leprosy incidence among close contacts for up to 6 years [1]. The Leprosy Post-Exposure Prophylaxis (LPEP) program demonstrated the feasibility of integrating contact tracing and SDR-PEP into routine activities in Tanzania and other countries. SDR-PEP administration was safe and well accepted by patients and healthcare workers [2]. WHO recommends systematic implementation of SDR-PEP in their global leprosy guidelines [3].

In 2021, the Tanzania’s Tuberculosis and Leprosy Programme (NTLP), with support of the Global Partnership for Zero Leprosy (GPZL), aligned its strategy with the WHO Global Leprosy Program [4]. This alignment was undertaken in accordance with the WHO 2021–2030 NTD Roadmap, which serves as the overarching policy instrument guiding global efforts to control and ultimately eliminate NTDs, including leprosy [5]. Tanzania’s updated leprosy strategy focuses on early detection, treatment, contact tracing, post-exposure prophylaxis, and the integration of leprosy services into primary healthcare [6]. Tanzania’s Zero Leprosy Action Plan (2022–2025) targets remaining leprosy challenges, including capacity building, surveillance, active case finding, stigma reduction, and resource mobilization [7]. The President’s Office – Regional Administration and Local Government (PO-RALG) coordinates health services at regional and local level, ensuring alignment with national policies and fostering effective delivery of community-based health programs [8].

Implementation of systematic contact tracing and SDR-PEP to reduce transmission of *Mycobacterium leprae* has challenges like lack of funding, trained personnel shortages, and constrained management capacity [9,10]. Integration of SDR-PEP into routine practice is still being tested. Multidrug therapy (MDT) is a cornerstone of the global leprosy elimination strategy. In Tanzania, Novartis supports the NTLP with MDT and SDR donations. The WHO Leprosy Elimination Monitoring Tool (LEMT) allows to follow trends towards transmission interruption by focusing on the number of new cases at sub-national level, especially child cases, because they indicate recent transmission of *M. leprae* [11].

The Morogoro Leprosy Elimination Program (MoLEP) aims to demonstrate *M. leprae* transmission interruption and long-term reduction of MDT needs, through comprehensive evidence-based interventions, which include contact tracing and SDR-PEP, and setting standards for replication.

## Process

### Project area

**Fig 1:**
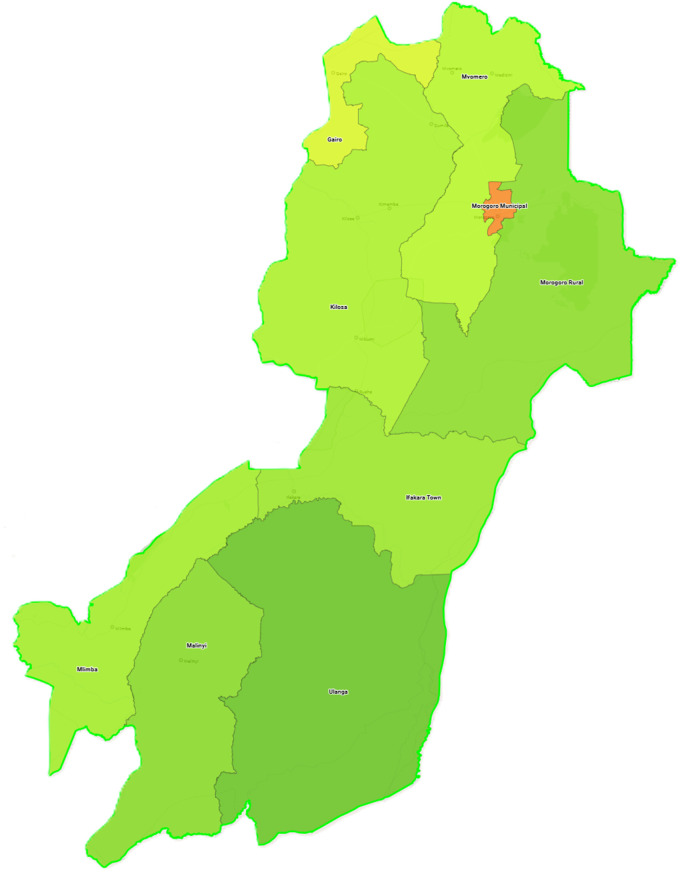
Map of Morogoro region displaying the nine councils (adapted from Thomas Brinkhoff: City Population, https://www.citypopulation.de/en/tanzania/admin).

Tanzania, one of WHO’s 23 global priority countries for leprosy, reported 1,454 new cases in 2023 [12]. Despite a robust leprosy elimination strategy and technical guidelines, resource constraints limit contact tracing and SDR-PEP implementation. MoLEP targets Morogoro, the region with the highest leprosy cases in the country. Morogoro has nine councils (Fig 1) and a population of about 3 million in 2024 [13]. In 2022, 246 cases (0.9 per 10,000) were registered, with 90.7% multi-bacillary, 2.0% child cases, 36.6% female cases, and 11.0% with grade 2 disability. The region has two leprosy centers: Nazareti Leprosy Center in Ifakara Town and another in Chazi, Mvomero.

### Partners

MoLEP activities are integrated into routine leprosy program structures, implemented by local health staff under PO-RALG, trained and following national guidelines, and reporting to NTLP through routine information systems. Strategic planning, technical and financial support, and progress monitoring are provided by: Novartis, the German Leprosy and Tuberculosis Relief Association (GLRA), and the Swiss Tropical and Public Health Institute (Swiss TPH). All collaborating partners have a longstanding history of advancing innovative leprosy control in Tanzania [14].

### Co-creation and planning

The design phase spanned over 18 months, with six months dedicated to defining scope and strategy, followed by 12 months of intensive partner discussions. Initial ideas emerged from exploratory meetings with NTLP and scientific partners, leading to the identification of an implementation partner. A detailed review of epidemiological data and available guidelines ensured alignment with global best practices and national structures. A three-day co-creation workshop brought together NTLP, regional representatives, and international partners, to discuss feasibility, resources, timelines and acceptability to ensure shared ownership and leveraging each partner’s expertise. This workshop succeeded in aligning all stakeholders and partners with the program’s objective (Table 1) and shape the first draft of the MoLEP protocol. The protocol was finalized through iterative feedback, to design a scientifically robust, contextually relevant, and operationally feasible protocol, aligning with global best practices and local needs. Furthermore, relevant training materials were identified or created for the capacity strengthening of the frontline health personnel.

**Table 1:** MoLEP aim and objectives.

**Aim** Demonstrate the feasibility of *M. leprae* transmission interruption and long-term reduction of MDT needs, through comprehensive evidence-based interventions, which include SDR-PEP, and setting standards for replication.
**Primary objectives**: Implement an integrated approach to interrupt *M. leprae* transmission in the Morogoro region: a. Strengthen health system capacity for leprosy in the Morogoro region b. Enhance early detection and post-exposure prophylaxis of leprosy contacts using SDR-PEP and establish related documentation (reporting and monitoring)
**Secondary objectives:** a. Develop an *M. leprae* transmission interruption implementation model that can be scaled up nationally and replicated in other endemic countries b. Operationalize the SDR donation model
**Explorative objectives:** a. Consider potential to integrate innovative approaches such as digital tools to support diagnosis and to streamline and standardize data collection; including the data of contacts for SDR-PEP b. Integrate antimicrobial resistance monitoring and infection status surveillance using rapid diagnostic tests (RDTs) c. Explore possibility of integration of AI (Artificial Intelligence) based EPCON model into data of Digital Health Information System 2 (DHIS2) portal which would help identify probable high-risk clusters (hotspots) for focused early screening and case detection campaigns

The MoLEP protocol is currently detailed for the first phase of implementation while defining the type of activities to be pursued over the following years of the planned project. The concept was presented to PO-RALG for implementation permit and introduction letter for the Morogoro region. The MoLEP protocol is seen as a working protocol and flexibility to integrate additional activities or adapt current practices based on emerging needs and evidence was explicitly stated. Annual review and planning workshops are held to define concrete activity plans for project years two to five. Following a formal launch in September 2024, the first trainings for health care professionals and community health workers was implemented based on a five-day and two-day intensive curriculum respectively, including both theoretical and hands-on leprosy training. The first annual review and planning meeting was held in June 2025.

## Core activities

**Fig 2:**
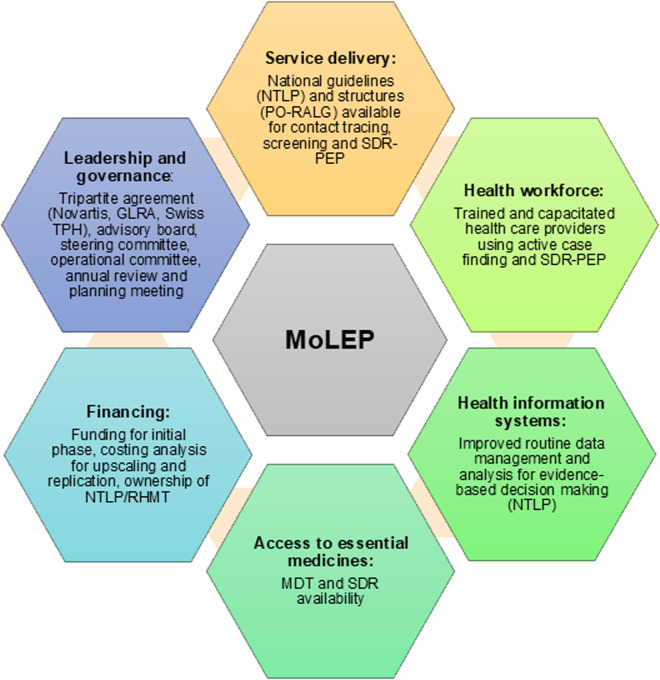
Core principles of the MoLEP program along the six building blocks of a health system (WHO [10]).

MoLEP focuses on integrating activities into the existing health system to promote local ownership and long-term sustainability, with a strong emphasis on capacity-building through structured training programs and systematic knowledge transfer (Fig 2). Routine data collection and supportive supervision serve as the foundation for improved monitoring, reporting, and evaluating progress, ensuring continuous evidence-based decision-making. Transparency, supported by a robust governance structure (tripartite agreement, advisory board, steering and operational committee), as well as through regular dissemination of results and operational experiences, serves to promote accountability, institutional learning, and facilitates scalability and replication in other settings.

Ensuring the uninterrupted availability of essential drugs like SDR and MDT is a prerequisite, requiring strengthened systems for procurement, supply chain management, and last-mile distribution.

**Fig 3:**
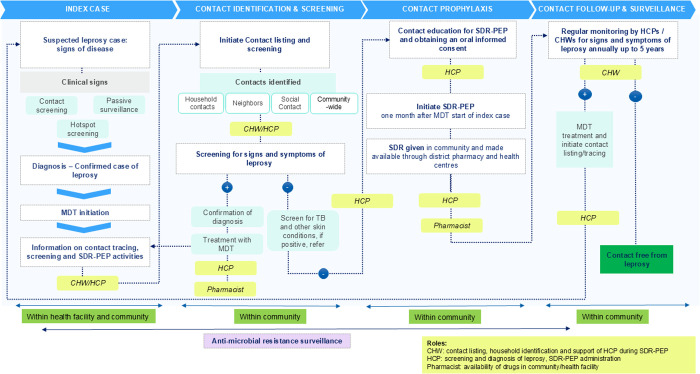
The journey of a leprosy contact using the MoLEP principles in Tanzania.

The project is being implemented initially in three councils, with a phased regional expansion to ultimately cover the entire Morogoro region. Key interventions include training sessions to build health worker capacity on routine contact tracing efforts, to identify and follow up on index cases while providing SDR to eligible contacts (Fig 3). In addition, catch-up (retrospective contact tracing) and acceleration (hotspot interventions, ensured drug supply) strategies address any gaps in leprosy case detection and treatment coverage (see next section).

### Accelerating *M. leprae* transmission interruption

Retrospective contact tracing involves the identification and follow-up of contacts of leprosy cases diagnosed in the past five years, aiming both, to clear the backlog of untraced contacts and to enhance early case detection. Following the completion of the leprosy training for health care professionals and community health workers, each health facility received a list of index cases in their catchment area to guide the systematic identification and follow-up of their contacts.

MoLEP uses a hotspot approach to cost-effectively target high-risk transmission areas. Hotspot communities are identified based on key epidemiological indicators including high detection rate of unrelated leprosy cases, the occurrence of child cases, and the presence of individuals with grade 2 disability [15]. In this context, “unrelated cases” refer to individuals diagnosed with leprosy who reside in different households and are not known close contacts of one another. These cases are presumed to derive from independent sources of exposure, indicating ongoing community-level transmission and potentially diverse circulating strains of *M. leprae*. Furthermore, intra-household cases are typically addressed through routine contact tracing activities. In addition to these quantitative indicators, the contextual knowledge and field experience of District Tuberculosis and Leprosy Coordinators are also considered when designating a community as a hotspot. Collectively, these factors signal ongoing transmission and identify potential gaps in healthcare access and capacity, thereby enabling focused interventions such as active case finding and SDR-PEP administration.

Strengthening the drug supply ensures consistent availability of MDT and SDR for effective leprosy management. NTLP orders SDR from WHO via a Novartis-subsidized system, while the Medical Stores Department handles in-country distribution. Regional stocks must reach pharmacies on time to support contact tracing efforts.

### Monitoring and evaluation

A robust monitoring and evaluation framework ensures routine data tracking, supportive supervision, quality assurance, and impact assessment to guide evidence-based decision-making. Quality assurance is maintained through continuous data quality evaluation, including monthly monitoring visits conducted by Regional and District Tuberculosis and Leprosy Coordinators (RTLC and DTLCs), which combine supportive supervision, data verification, and on-site training. In addition, monthly data review and feedback are conducted by the MoLEP operational committee, while quarterly steering committee meetings provide a platform for strategic oversight and planning.

To track progress toward the interruption of transmission, the WHO-recommended LEMT is regularly updated. Program impact is assessed using key indicators, including the new autochthonous case detection rate, prevalence of child cases, proportion of cases with grade 2 disability, number of contacts receiving SDR-PEP, decline in the number of identified hotspots over time, and the number of health staff capacitated. Together, these mechanisms ensure a responsive and adaptive implementation strategy aligned with national and global leprosy elimination goals.

A comprehensive cost assessment evaluates expenses of all program components including training, contact tracing, screening, and reporting and thereby informing resource allocation and sustainability. It guides planning, investment priorities, scaling and replication, ensuring transparency and supporting long-term leprosy elimination efforts.

MoLEP includes antimicrobial resistance monitoring using skin biopsy specimens from the border of an active lesion, in a subsample of MDT-treated patients, leveraging from experiences of prior studies in Tanzania [16]. Infection monitoring using a lateral flow assay (LFA) to quantitatively detect IgM against *M. leprae*-specific phenolic glycolipid I (anti-PGL-I) based on up-converting reporter particles (UCP) and immunochromatography, also known as the UCP-LFA test platform [17], will expand impact assessment to carriers and asymptomatic cases. Infection monitoring is conducted periodically among children to document recent transmission of *M. leprae* and changes therein between MoLEP and non-MoLEP districts. This will allow the monitoring of the transmission reduction in intervention districts. Relevant tools have recently been developed, while implementation protocols are still needed.

### Governance and dissemination

The international partners Novartis, GLRA and Swiss TPH have a tripartite agreement outlining their roles and responsibilities. GLRA has a long-standing agreement with the Tanzanian MoH to support leprosy and TB control. MoLEP is supported by a governance structure, with an operational committee for day-to-day management and implementation, and a steering committee providing strategic oversight and guiding high-level decision-making. An advisory board including members of RHMT, NTLP, WHO, NIMR, and the tripartite partners will assess the programs process bi-annually (Fig 2). During annual meetings, implementation progress, challenges, and lessons learned, facilitating planning and protocol adjustments as needed.

MoLEP results will be shared globally through conferences, workshops, and peer-reviewed publications to promote evidence-based leprosy elimination strategies. Mid-term (2027) and endline (2029) evaluations will assess progress, effectiveness, and sustainability, offering recommendations to refine the program and inform global strategies.

## Relevance

Public–private partnerships (PPPs) effectively address complex health challenges by combining the public sector’s focus on equity and regulation with the private sector’s efficiency, innovation, and resource mobilization. PPPs drive innovation while prioritizing public health goals [18,19]. For example, a PPP in Kenya, rapidly enhanced the public health sector’s COVID-19 response through coordinated private sector efforts [20]. However clear governance, transparency, and mutual accountability are essential to mitigate potential conflicts of interest [21], as recognized by stakeholders in Tanzania [22].

Co-creation in public health fosters local alignment, ownership, and applicability. Although time-consuming, it integrates diverse stakeholder perspectives to address local needs effectively [23]. This participatory approach enhances intervention relevance, stakeholder commitment, and accountability [24], while promoting mutual learning, power-sharing, and practical community solutions [25].

Expanding MoLEP activities beyond Morogoro could accelerate leprosy elimination in Tanzania. Neighboring regions can adapt interventions to their context, such as leprosy epidemiology, infrastructure and health services. National scale-up of MoLEP activities would strengthen progress toward WHO leprosy targets (reduction in new case detection rate, reduction in grade 2 disability, reduction in child cases). These targets are nested within the broader strategic vision of achieving zero infection and disease, zero disability, and zero discrimination, and serve as critical benchmarks for measuring progress toward leprosy elimination at both national and global levels.

MoLEP relies on basic routine health system structures and capacities, such as active case finding, diagnosis and treatment, as well as community engagement. In low-endemic areas, adaptions may be required to enhance surveillance, including molecular tools for transmission monitoring, to detect sporadic cases and prevent disability. Moreover, economic feasibility must be considered, balancing leprosy vigilance with integration into broader skin health programs. Contextual factors, such as cultural perceptions [2] and health system priorities should also be addressed to ensure sustainability and acceptance.

MoLEP will generate crucial evidence on strategies for accelerating *M. leprae* transmission interruption through an integrated approach that combines systematic contact tracing, chemoprophylaxis using SDR-PEP, and targeted interventions, all embedded within a robust monitoring and evaluation framework. Its modular and adaptable design enables context-specific implementation and scalability thereby reinforcing health system capacities for the early detection and prevention of leprosy. Through the integration of operational research and the promotion of evidence-informed decision-making, facilitated by capacity strengthening, supportive supervision, and high-quality data systems, MoLEP enhances the effectiveness of leprosy control efforts. This approach provides a sustainable and equity-oriented pathway to interrupt *M. leprae* transmission, adaptable across diverse epidemiological settings and health system contexts.

## Abbreviations

LEMT: Leprosy Elimination Monitoring Tool
LPEP program: Leprosy Post-Exposure Prophylaxis program
MDT: Multidrug Therapy
MoLEP: Morogoro Leprosy Elimination Program
PEP: Post-Exposure Prophylaxis
PPP: Public–Private Partnership
SDR: Single-Dose Rifampicin
